# The impacts of thermal heterogeneity across microhabitats on post-settlement selection of intertidal mussels

**DOI:** 10.1016/j.isci.2023.108376

**Published:** 2023-10-31

**Authors:** Yue Tan, Yong-Xu Sun, Ya-Jie Zhu, Ming-Ling Liao, Yun-Wei Dong

**Affiliations:** 1 The Key Laboratory of Mariculture, Ministry of Education, Fisheries College, Ocean University of China, Qingdao 266003, P.R. China; 2 State Key Laboratory of Marine Environmental Science, College of Ocean and Earth Sciences, Xiamen University, Xiamen 361102, China

**Keywords:** Marine organism, Environmental science, Aquatic science, Zoology

## Abstract

Rapid genetic selection is critical for allowing natural populations to adapt to different thermal environments such as those that occur across intertidal microhabitats with high degrees of thermal heterogeneity. To address the question of how thermal regimes influence selection and adaptation in the intertidal black mussel *Mytilisepta virgata*, we continuously recorded environmental temperatures in both tidal pools and emergent rock microhabitats and then assessed genetic differentiation, gene expression patterns, RNA editing level, and cardiac performance. Our results showed that the subpopulations in the tidal pool and on emergent rocks had different genetic structures and exhibited different physiological and molecular responses to high-temperature stress. These results indicate that environmental heterogeneity across microhabitats is important for driving genetic differentiation and shed light on the importance of post-settlement selection for adaptively modifying the genetic composition and thermal responses of these intertidal mussels.

## Highlights

• Intertidal species are subject to rapid selection under environmental stress
• Summer high temperature is essential in promoting rapid genetic selection
• Post-settlement selection can modify genetic structure across microhabitats

## Graphical abstract

**Figure:**
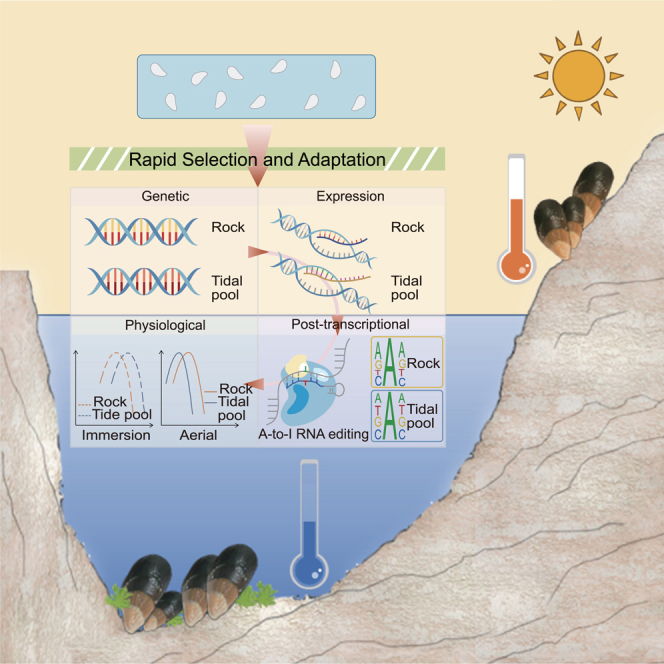

## Introduction

Climate change is affecting almost all marine organisms,^1^^,^^2^^,^^3^ and assessing and predicting the vulnerability of species to present and future warming are an important challenge.^4^^,^^5^ Natural populations face a wide range of thermal environmental conditions, even across short distances on the landscape. The term “refugia” denoting habitats with minimal influence from climate change can induce optimal natural selection for different local traits in each habitat.^6^ Under intense selection pressure, genetic evolutionary adaptation has the potential to provide a buffer against extinction or migration due to climate change and become an important factor in predicting species’ vulnerability to climate-driven decline or extinction.^7^

Analyses of global warming’s effects on natural populations must be developed in a broad ecological and evolutionary context. A fundamental focus of ecology and evolutionary biology is to determine if and how natural populations adapt to rapid environmental change.^8^ Abundant evidence has accumulated over the past several decades showing that organisms may accumulate genetic variations within a few generations and subsequently undergo rapid adaptation to local environmental demands through the process of natural selection within a short time frame.^9^^,^^10^^,^^11^ Genetic variation is considered to be the most important component of adaptive evolution.^12^ The classical genetic theory assumes that genetic variation driven by environmental selection pressures generally occurs over timescales of many generations and assumes that most genetic variation has a limited adaptive effect.^13^ However, recent advances in molecular genetics and bioinformatics have allowed the detection of signatures in genomic data that identify recent adaptive events, thereby enabling investigators to demonstrate the importance of rapid genetic evolution.^14^^,^^15^ Many studies have now demonstrated rapid changes in heritable traits and shown that evolutionary adaptation will occur rapidly when natural selection is strong.^16^^,^^17^^,^^18^ Furthermore, recent work has revealed that the rapid adaptation of species to the environment may involve only a few genetic loci.^19^ Populations have evolved adaptations to tolerate or even take advantage of potentially stressful changes in the environmental conditions they experience. The strong link between environment and rapid genetic evolution is significant because it may allow the maintenance of the stability of populations and communities, as well as the generation and maintenance of diversity within and among populations.^20^ Although the dynamics and feedback of these rapid genetic adaptations have now been demonstrated many times, we are still far from understanding the temporal and spatial scales of environmental variation, notably in temperature, that occur in nature. Likewise, the types of physiological and molecular processes that benefit from rapid genetic change remain to be fully elucidated.

Among various molecular alterations that could play significant roles in enhancing environmental tolerance, one noteworthy process is RNA editing.^21^ The debate regarding whether RNA editing primarily signifies genetic adaptation through natural selection or rather reflects a form of environmental-induced plasticity underscores the complexity of its potential implications.^22^ RNA editing is an evolutionarily conserved post-transcriptional modification that can lead to amino acid recoding and altered protein function.^23^ A-to-I (A > I) and C-to-U (C > U) are the two most common forms of RNA editing, accomplished by the enzymes Adenosine Deaminase Acting on RNA (ADAR) and Adenosine-5′-*O*-phosphate-beta-ethyl-esterase-cytidine-3′-monophosphate-deaminase (APOBEC), respectively.^24^ Changes in A-to-I RNA editing can alter codon properties or base-pairing interactions within the RNA structure and contribute to the regulation of post-transcriptional gene expression levels. Editing of precursor messenger RNAs (mRNAs) can create or disrupt splice sites, while the biogenesis and processing of microRNAs and the sequence and context of their targets are also affected by A-to-I editing. RNA editing that leads to protein recoding plays an important role in development and in helping organisms adapt to environmental changes.^25^ Recent studies show that the evolutionary acquisition of A > I RNA editing sites can facilitate temperature adaptation in octopuses and *Drosophila*.^26^^,^^27^ However, whether RNA editing could respond rapidly to environmental stressors, especially in natural populations, remains underexplored.

Certain environmental features that may be important in driving adaptive genetic variation are also inadequately understood. An important but easily overlooked aspect in assessing the role of adaptive genetic selection is the ecological impact of spatial variation across microhabitats.^28^^,^^29^^,^^30^ Adaptive genetic selection occurs when populations evolve traits that confer higher fitness in the local environment than in foreign environments, regardless of distance.^31^^,^^32^ Many studies have highlighted the important role of small-scale genetic variation,^33^^,^^34^ where genetic adaptation occurring at small scales generates genetic diversity that helps populations survive when major environmental changes occur within a local area.^35^ Therefore, integrated consideration of rapid genetic adaptation across small-scale microhabitats is essential for assessing and predicting the effects of global warming on species.

The rocky intertidal zone presents an opportune system for elucidating the adaptive strategies employed by organisms to mitigate the challenges posed by thermal extremes.^36^^,^^37^ The intertidal temperature environment is highly variable at multiple temporal and spatial scales.^38^^,^^39^^,^^40^ Sessile species with pelagic larval stages offer especially powerful experimental systems for addressing microhabitat-specific adaptations. During settlement, larvae attach randomly to substrates in tidal pools and tidally emerged rock, leading to populations of adults that encounter very different physical, physicochemical, and environmental conditions. Tidal pools are shallow seawater pools that remain as distinct bodies of water during low tide,^41^ and they usually maintain lower temperatures compared to the sun-exposed rock microhabitats in summer; as a result, they act as a protective haven, providing intertidal organisms with a refuge from the harsh impacts of extreme heat stress.^42^ Environmental heterogeneity in small scales can put highly variable selection pressure on species.^34^ Although research on the integration of biological physiological diversity, genetic adaptation, and microhabitats has received increasing attention and made some progress in recent years,^30^^,^^43^^,^^44^ the current state of knowledge regarding the roles of rapid genetic responses to environmental heterogeneity necessitates further exploration.

An excellent study organism for these purposes is the black mussel, *Mytilisepta virgata*.^45^ This mussel is a dominant sessile species on the subtropical and tropical coasts of the Indo-West Pacific. In this broad region, the species occupies a wide range of microhabitats, including tidal pools and sun-exposed and shaded rocky sites in the mid to high intertidal zone.^30^ The high mortality of this species in sun-exposed environments during summer implies that it is highly sensitive to extremes of high temperatures.^46^^,^^47^^,^^48^ It is important to note that the black mussel, unlike economically exploited bivalve species, is not typically utilized as a food product. As such, it is less likely to be affected by the development of aquaculture and fisheries. This unique attribute positions the black mussel as an ideal candidate for studying the mechanisms of thermal adaptation in wild populations. Many intertidal bivalve species have developed various mechanisms to cope with high temperatures.^49^^,^^50^ To examine how the black mussel varies genetically, molecularly, and physiologically across microhabitats, we first identified genome-wide single-nucleotide polymorphisms (SNPs) for calculating genome-wide genetic structure and determining whether genetically distinct conspecifics occurred in different microhabitats, likely due to post-settlement selection driven by environmental, e.g., temperature differences. We then determined gene expression responses to high temperature between two microhabitat-specific populations using RNA sequencing (RNA-seq), characterized the responses of heart rate to temperature in these populations, and examined how temperature affected levels of RNA editing in the different groups. By conducting this broad set of analyses, we aimed to answer two primary questions: (1) can the black mussels exhibit rapid post-settlement strong selection, driven by environmental stress even at small spatial scales, resulting in distinct genetic structures? and (2) to what extent do exceptionally high summer temperatures, potentially reaching 60°C, contribute to the adaptive physiological and molecular responses in the black mussels?

## Results

### Thermal environments in different microhabitats

**Figure 1:**
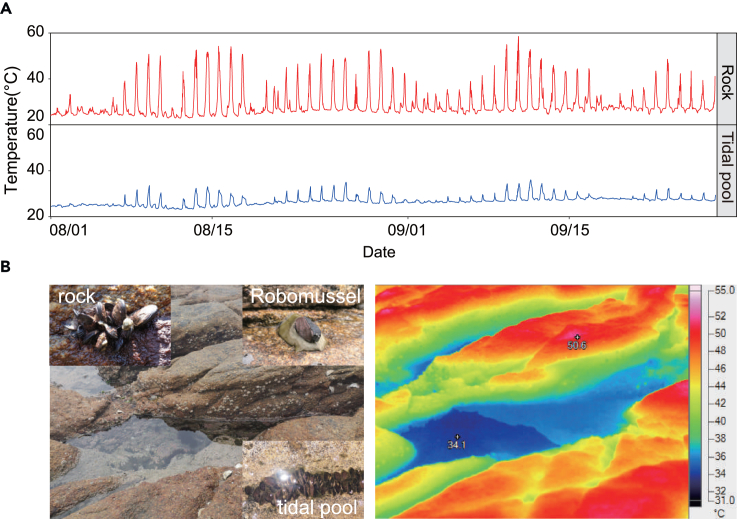
Thermal environments in different microhabitats (A) *In situ* operative temperature of different microhabitats from July to September 2020 obtained with Robomussels. (B) Temperature variation across microhabitats in the intertidal zone determined by an infrared camera.

Thermal environments on the shore varied considerably among different microhabitats (tidal pool vs. emergent rock) and showed a high degree of spatial and temporal heterogeneity. Thermal environments on the shore showed distinct daily fluctuation from August to September 2020 (Figure 1). During the period of measurement, the average temperature of the tidal pool was 26.62°C ± 1.94°C and the average temperature of the rock was 28.80°C ± 6.07°C. From July to September 2020, the values of T_99_ for tidal pool and rock microhabitats were 33.16°C and 51.69°C, respectively.

### Population genetic structure and phylogenetic analysis

#### Non-neutral variation

**Figure 2:**
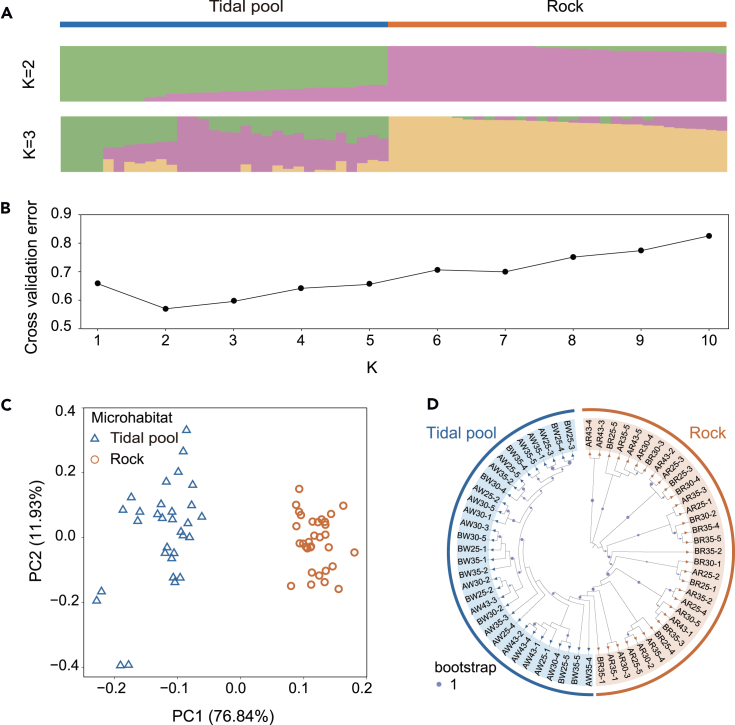
Population genetic structure and phylogenetic analysis of non-neutral variation (A) Population structure of *Mytilisepta virgata* in tide pools and rock microhabitats. Cross-validation (CV) errors suggest that the 63 individuals were divided into 2 genetic populations. (B) Population structure analysis. Each individual is represented by a vertical bar that is divided by K-colored segments representing the likelihood of membership to each cluster. (C) Clustering of *M. virgata* populations based on principal-component analysis (PCA). Each point represents an individual and is colored according to the collection site. (D) Approximate maximum likelihood tree of 63 *M virgata* accessions based on genetic distance. Color bars indicate accessions with tidal pool and rock microhabitats. The size of the circular symbol indicates the magnitude of the bootstrap value, with a circle of a specific size in the illustration serving as a reference control for the value of 1.

To understand the population genetic relationships among all individuals from different microhabitats, the number of subgroups (K value) was preset to 1–10 for clustering; the clustering results were cross-validated, and the optimal number of clusters was determined according to the lowest cross-validation error (CV error) rate. In this study, CV error ranged from 0.5699 to 0.8255. We mainly focused on two cases (K value = 2 and K value = 3), because they clearly showed the major structure of two populations. It can be clearly shown that the tidal pool individuals and the rock individuals were divided into two groups (Figure 2A), when the CV error was 0.5699 and the optimal K = 2 (Figure 2B). With an increase of values of K (K = 3), the 63 individuals from different microhabitats were split into three groups (Figure 2A). Although there were also a large number of genotype admixtures, a certain genetic structure was found between different populations. We finally concluded that K = 2 was the most suitable number of genetic clusters based on the observation that experimental populations’ distributions corresponded spatially with major geographical features.

To better understand the genetic structure, a principal-component analysis (PCA) was applied based on identified SNPs among all 63 individuals. PCA analysis generally confirmed the pattern of the genetic structure obtained with K = 2. Our results showed two principal components accounted for 76.84% (PC 1) and 11.93% (PC 2) of the total variability (Figure 2C). The results of PCA analyses showed genetic clusters that are similar to those obtained by genetic structure analyses; both analyses showed a clear divergence between groups. Moreover, compared to rock microhabitat individuals, the more discrete “cluster-like” population structure of individuals in tide pools implied this population has higher individual variations. Tidal pool individuals were more dispersed in both genetic structure and PCA analysis, with more dispersed SNP loci within the group; the high dispersion of tidal pool individuals compared to other populations implies that the population is more heterogeneous.

To better visualize the relationship between sample distribution and genetic structure, a phylogenetic tree of 63 individuals was constructed (Figure 2D). The individuals of each population cluster together, and there are clear genetic boundaries between different populations. Although there was some genetic differentiation within each group, the tidal pool and rock microenvironment groups were separated according to the evolutionary tree. The AMOVA analysis results indicated that 76.03% of the total genetic variation was attributed to within-microhabitat genetic diversity, while the remaining 23.97% was ascribed to genetic differentiation among microhabitats (Table S1).

#### Neutral variation

**Figure 3:**
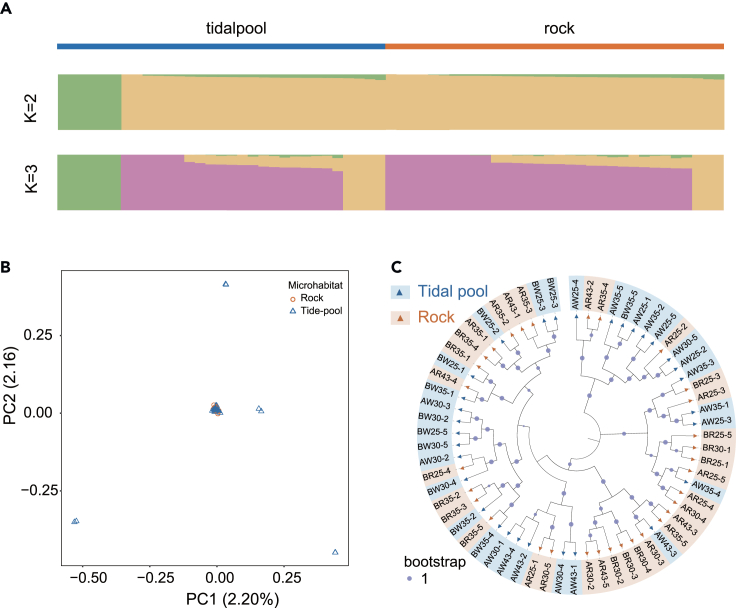
Population genetic structure and phylogenetic analysis of neutral variation (A) Population structure of *Mytilisepta virgata* in tidal pools and rock microhabitats. (B) Clustering of *M. virgata* populations based on principal-component analysis (PCA). Each point represents an individual and is colored according to the collection site. (C) Approximate maximum likelihood tree of 63 individuals based on genetic distance. The size of the circular symbol indicates the magnitude of the bootstrap value, with a circle of a specific size in the illustration serving as a reference control for the value of 1.

The genetic structure of the neutral loci showed that mussels from the two microhabitats were genetically more similarly distant (Figure 3A). Notably, when the genetic structure of K = 2 and K = 3, a small proportion of individuals in the tide pool subpopulation (the green cluster) had a significantly different genetic structure from the others. PCA clustering indicated that most individuals in both tide pool and rock clustered together, but several tide pool individuals were dispersed (Figure 3B). The tree of neutral loci further indicates that the two subpopulations of tide pools and rocks were genetically more similarly distant (Figure 3C).

#### Heterozygosity

The heterozygosity of non-neutral loci was higher than that of neutral loci (Table 1). The heterozygosity of neutral loci was significantly greater for the rock subpopulation than for the tidal pool; on the contrary, the heterozygosity of the non-neutral loci of the rock subpopulation was significantly lower than that of the tidal pool subpopulation.

**Table 1:** Heterozygosity of neutral and non-neutral loci in tidal pools and rock subpopulations

Heterozygosity			
	Rock	Tidal pool	p value
Neutral variation	0.0587 ± 0.0611	0.0358 ± 0.0565	0.0667
Non-neutral variation	0.1254 ± 0.6333	0.7689 ± 0.7880	0.0060

### Differentially expressed genes (DEGs)

**Figure 4:**
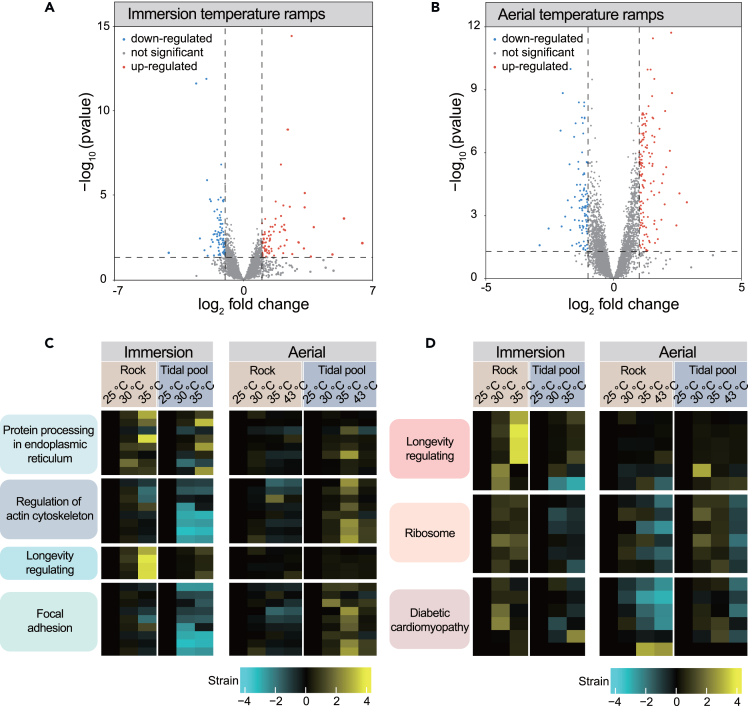
Differentially expressed genes (DEGs) and KEGG enrichment analysis DEGs over temperature stress across microhabitats populations in immersion treatment (A) and aerial treatment (B). The red dots and blue dots represent significantly upregulated and downregulated genes, respectively, and the gray dots represent genes whose expressions did not change significantly. (C) KEGG pathways enriched to differential genes in tide pools and rocks in immersion temperature ramps. (D) KEGG pathways enriched to differential genes in tide pools and rocks in aerial temperature ramps.

Integrated analysis of DEGs was analyzed using the R project Deseq2 package (p < 0.05 and |log2FC| > 1.0). Significantly differentially expressed genes between different microhabitats were generated as shown in the volcano plot; the red dots represent significantly upregulated genes, whereas the blue dots represent significantly downregulated genes (Figures 4A and 4B).

In immersion temperature ramps, the expression of 172 genes was significantly altered between tidal pools and rock (Figure 4A). Compared to the tidal pool subpopulation, 85 genes and 87 genes in the rock subpopulation were upregulated and downregulated, respectively. Among the upregulated genes, heat shock protein beta-6 (HspB6), Rho-associated protein kinase 2 (ROCK2), fibroblast growth factor receptor 2 (FGFR2), crystallin alpha B (CRYAB), and heat shock protein Hsp-16.1 (Hsp-16.1) exhibited notable increases in gene expression. Downregulated genes encompassed Heat shock 70-kDa protein 5 (Hspa5), transitional endoplasmic reticulum ATPase (VCP), Ubiquitin-conjugating enzyme E2 J1 (UBE2J1), protein transport protein SEC31 (SEC31B), S2P endopeptidase (S2P), Mannose-binding lectin (MBL), cell division control protein 42 (Cdc42), NCK-associated protein 1 (NCKAP1), Integrin alpha9 (ITGA9), dedicator of cytokinesis protein 1 (DOCK1), Baculoviral IAP Repeat Containing 3 (BIRC3), collagen type VI alpha 3 (COL6A3), and Caveolin-1 (CAV1). The genes exhibiting differential expression between tidal pools and rock across different temperatures were enriched in “Protein processing in endoplasmic reticulum”, “Regulation of actin cytoskeleton”, “Longevity regulating,” and “Focal adhesion” pathways (Figure 4C).

In aerial treatments, a total of 231 genes as DEGs were identified; compared to the tidal pool subpopulation, 133 genes and 98 genes in the rock subpopulation were upregulated and downregulated, respectively. Among the upregulated genes, heat shock protein beta-6 (HspB6), heat shock protein Hsp-16.1 (Hsp-16.1), alpha-crystallin B chain (CRYAB), adenylate cyclase, Ribosomal proteins (such as L9e, SAe, L32e, S10e, L27e, L34), NADH dehydrogenase (ubiquinone) Fe-S protein 4 (Ndufs4), ubiquinol-cytochrome c reductase subunit 6 (QCR6), NADH dehydrogenase (ubiquinone) 1 alpha subcomplex subunit 5 (NDUFA5), Type III collagen alpha (COL3A1), and Ras-related C3 botulinum toxin substrate 1 (RAC1) exhibited notable increases in expression. Conversely, downregulated genes encompassed solute carrier family 2 (Slc2a1). The genes exhibiting differential expression within tidal pools and rock across the temperature were enriched in “Longevity regulating”, “Ribosome,” and “Diabetic cardiomyopathy” pathways (Figure 4D).

### RNA editing analysis

Given that A-to-I RNA editing exists in all metazoans,^51^ the analysis in this study focused exclusively on A-to-I RNA editing in different subpopulations inhabiting different microhabitats. A-to-I RNA editing is prevalent in *M. virgata*, and the percentage of A-to-I RNA editing varied among microhabitat groups, different temperatures, and different treatments. We did not examine the amounts of recoding due to RNA editing, albeit this could be an important contributor to rapid adaptive change.

**Figure 5:**
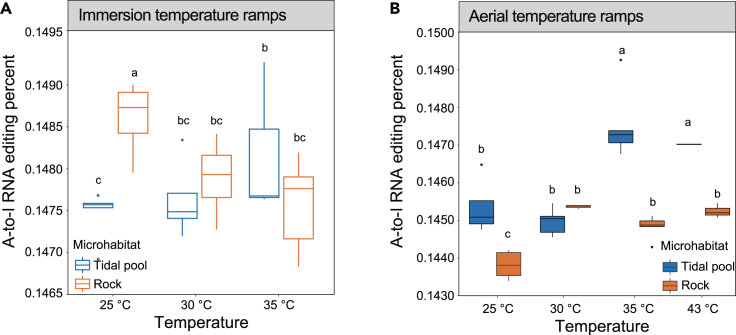
RNA editing analysis of mussels from different microhabitats (A) The proportion of A-to-I RNA editing of mussels in tidal pools (blue box) and rock (yellow box) under immersion treatment. (B) The proportion of A-to-I RNA editing of mussels in tidal pools (blue box) and rock (orange box) under aerial treatment. Means with the same letter did not significantly differ at p < 0.05, according to the general linear model (GLM).

In the immersion treatment, the percentage of A-to-I RNA editing sites was significantly higher for individuals on rocks than those from tidal pools at 25°C. The percentage of A-to-I RNA editing sites was lower at 30°C and 35°C than at 25°C in the rock subpopulations; the percentage of A-to-I RNA editing sites was higher at 30°C than 25°C and 35°C in the tidal pool subpopulations (Figure 5A).

In the aerial treatment, the percentage of A-to-I RNA editing sites was significantly higher for individuals in the tidal pool subpopulation than the rock subpopulation except at 30°C. For the mussels on the rocks, the percentage of A-to-I RNA editing sites was significantly higher at 30°C, 35°C, and 43°C than at 25°C; similarly for the mussels in the tidal pool, the percentage of A-to-I RNA editing sites was significantly higher at 35°C and 43°C than 25°C and 35°C (Figure 5B). In both microhabitats, the RNA editing levels in the immersion treatment were significantly higher than those in the aerial treatment under equivalent temperature conditions (Figure S1).

### Cardiac performance

In the immersion treatment, the heart rates of mussels in tide pools at 25°C, 30°C, and 35°C were 31.33 ± 3.24, 41.24 ± 6.80, and 67.25 ± 7.22 beats min^−1^ (mean ± SE), respectively. In contrast, mussels on rocky substrates at the same temperatures exhibited heart rates of 26.31 ± 4.22, 36.46 ± 5.80, and 60.26 ± 7.05 beats min^−1^, respectively. In the aerial treatment, mussels in tide pools at 25°C, 30°C, 35°C, and 43°C had heart rates of 4.20 ± 1.10, 7.24 ± 1.96, 8.12 ± 2.34, and 10.87 ± 0.95 beats min^−1^, respectively. Meanwhile, mussels on rocky substrates at the corresponding temperatures displayed heart rates of 6.08 ± 0.57, 7.65 ± 1.04, 10.11 ± 2.50, and 12.74 ± 2.10 beats min^−1^, respectively.

**Figure 6:**
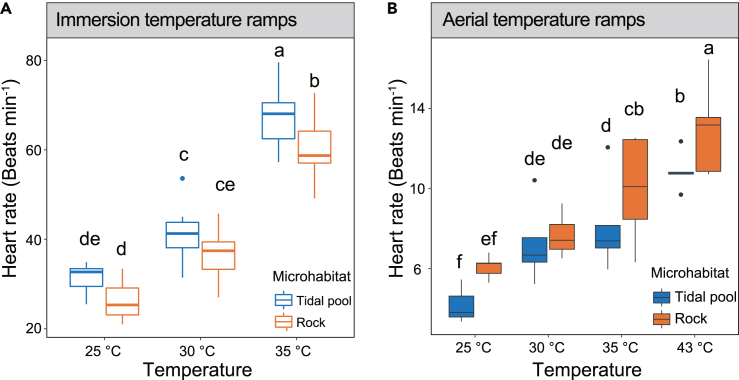
Cardiac performance of mussels from different microhabitats Heart rate of mussels in immersion treatment (A) and aerial treatment (B). The individuals from tidal pool and rock habitats are indicated in blue and orange. Means with the same letter did not significantly differ at p < 0.05, according to the general linear model (GLM).

The response pattern of increased heart rate with increasing temperature was consistent for different microhabitats in the immersion and aerial treatments, but *M. virgata* in tidal pools and rocks exhibited different patterns in the face of temperature changes. Firstly, in both the rocks and the tidal pools, the heart rate of *M. virgata* in the immersion treatment was significantly higher than that in the aerial treatment under equivalent temperature conditions (Figure S2). Secondly, in the immersion treatment, the heart rates of tidal pool and rock individuals were not significantly different at either 25°C or 30°C, while the heart rates of tide pool individuals were significantly higher than those of rock individuals at 35°C. The effect sizes for microhabitat and temperature were 0.201 and 0.874, respectively; in the aerial treatment, there were no significant differences between tidal pool and rock individuals at 25°C and 30°C, but heart rates of rock individuals were significantly higher than those of tidal pool individuals at 35°C and 43°C. The effect sizes for microhabitat and temperature were 0.174 and 0.665, respectively. Finally, in immersion temperature ramps, the heart rates of the black mussels on rocks and in tidal pools exhibited a significant increase with rising temperatures. In aerial temperature ramps, the black mussels on rocks showed a significant increase in heart rate with rising temperatures starting at 30°C, whereas the black mussels in tide pools showed a significant increase in heart rate as temperatures increased (except between 30°C and 35°C) (Figure 6).

## Discussion

Rapid temperature-adaptive genetic selection may be essential for enabling natural populations to cope with extreme thermal environments. Even in a relatively short time, rapid adaptive selection can filter individuals living in different microhabitats into genetically distinct populations with different responses to temperature. This study demonstrated that *M. virgata* inhabiting tidal pool and rock microhabitats exhibited physiological, genetic, and molecular differences in thermal responses after summer heat selection. Our results provide further proof that genetic adaptations generated by small-scale microenvironments aid populations in surviving during hot summers when significant environmental changes occur within localized areas. Thermal heterogeneity at the microhabitat scale and rapid microevolutionary reactions thus should be taken into account when assessing and predicting the vulnerability of organisms to present and future warming.

### Rapid genetic selection and adaptation occurred across microhabitats

Organisms may rapidly evolve in response to strong selection pressure, and the maintenance of phenotypic and genetic variation by temporally variable selection is observed in a variety of organisms.^52^ Extreme heat is one of the most damaging threats to marine life and can act as a significant selection pressure. In the present study, after high summer temperature selection, the black mussels were quickly filtered into two subpopulations based on the tidal pool and emergent rock microhabitats.

Considerable adaptive variability in cardiac thermal tolerance was found between tide pool and rock microhabitats in black mussels. Individuals on the rocks had higher cardiac heat tolerance when exposed to high temperatures in the air, whereas individuals in the tidal pools had higher cardiac heat tolerance at high temperatures during immersion. This implies physiological adaptation to the local thermal environment. The structured rocky shores of the intertidal zone create a large difference in environmental temperatures between the two microhabitats, with extreme heat stress (i.e., T_99_) being significantly lower in the tide pools than in the rock microhabitat.^53^ This difference in thermal stress has a significant impact on thermal physiological response and induces different cardiac functional responses between the two microhabitats. Organisms can rapidly regulate gene expression to alleviate environmental stresses.^54^ Environmental stress activates signal transduction pathways in cells, thereby regulating pathway-related gene expression and metabolism.^55^ In the present study, many DEGs were significantly enriched in the “Protein processing in endoplasmic reticulum” category in the immersion treatment. An endoplasmic reticulum stress response is activated for cellular self-protection when the dynamic balance within cells is perturbed, for example, by nutrient deficiency, imbalance in calcium ion metabolism, toxin stimulation, and sustained oxidative stress.^56^ Extreme heat can also cause protein denaturation and promote expression of genes in the category “Protein processing in the endoplasmic reticulum” such as those encoding heat shock proteins (Hsp) and cuticle proteins.^57^^,^^58^

Heat shock proteins involved in the “Longevity Regulating Pathway” have long been used as indicators of heat sensitivity. Hsp family genes are involved in multiple cellular metabolic pathways and play a critical role in maintaining cellular homeostasis, including protein folding, assembly, and degradation, and transcription.^59^^,^^60^ Studies have shown that organisms inhabiting different microhabitats have different patterns of Hsp70 expression.^61^^,^^62^ In mussels, the expression of heat shock protein genes increased with increasing temperature, suggesting that temperature has a strong impact on the biological functions of mussels. In the present study the expression pattern of heat shock protein genes varied among treatment groups, with much greater interpopulation difference in the aerial treatment than in the immersion treatment. These findings support the conjecture that microhabitat heterogeneity favors thermal adaptation of mussels to their local habitat conditions.

Studies suggest that RNA editing is sensitive to temperature change.^27^^,^^63^ The multifaceted relationship between RNA editing and thermal adaptation in *M. virgata* is elucidated by several key findings in this study. Firstly, the differential RNA editing patterns observed at varying temperatures across organisms suggest that RNA editing is a dynamic mechanism of gene regulation for rapid adaptation to different thermal environments,^64^ highlighting the remarkable versatility of RNA editing as an adaptive response. This adaptability enables organisms to intricately adjust their molecular machinery in response to the temperature. Secondly, the alteration patterns in RNA editing levels vary in response to heat stress among distinct subpopulations inhabiting diverse microhabitats; in immersion temperature ramps *M. virgata* in the tidal pool appears to exhibit higher levels of RNA editing than individuals in the emergent rock habitat, implying a crucial role of environmental conditions in molding RNA editing profiles. These observations underscore the remarkable adaptive flexibility of organisms, with distinct RNA editing patterns potentially bestowing selective advantages within specific microhabitats. Furthermore, RNA editing levels demonstrated a notable increase under identical temperature conditions in immersion temperature ramp than aerial temperature ramp. This contrast can be ascribed to the restricted availability of oxygen during aerial temperature ramps, potentially inducing anaerobic respiration.^65^ Organisms modulate A-to-I RNA editing as part of their molecular repertoire for coping with hypoxic stress. This adaptive response, in turn, is likely to enhance the organism’s capacity to acclimatize to elevated temperatures. In summary, our study reveals a multifaceted relationship between RNA editing and thermal adaptation, highlighting the pivotal role of RNA editing as a versatile adaptive mechanism in response to environmental thermal challenge. This elucidation not only contributes to a deeper understanding of the molecular underpinnings of thermal adaptation but also underscores the significance of RNA editing in organisms' dynamic responses to environmental thermal challenges.

In our experiments, we did not evaluate the relative levels of synonymous and nonsynonymous (recoding) editing of mRNAs. Protein-recoding mRNA editing plays an important role in helping organisms adapt to environmental changes,^66^^,^^67^ so further study of the *M. virgata* populations is warranted to determine the extent of protein variation induced by mRNA editing.

### Adaptive genetic differentiation among microhabitats

Environmental heterogeneity is commonly associated with genetic heterogeneity, as shown by Antonovics^68^ and Hedrick.^69^ Even at distances of a few meters, genetic differentiation can be observed, which is typically caused by the physical or biological characteristics of the environment, or a combination of both.^70^ For example, due to the extensive dispersal potential of planktonic larvae in the marine environment, gene flow among black mussel populations in different microhabitats becomes feasible; black mussels with an identical origin attach randomly to tidal pool surfaces and rocks that are exposed during low tide and thereby encounter different environmental thermal stress. These differences in thermal conditions can lead to post-settlement genetic differentiation among individuals inhabiting different microhabitats.

Analysis of neutral loci revealed no excessive genetic variation between microhabitats, and neutral loci remained largely similar in both populations, suggesting that the two subpopulations, tide pools and rocks, share common parental populations. However, adaptive genetic differences were observed between the tide pool and rock microhabitats of black mussels in the non-neutral loci. Rock individuals subjected to high temperature selection possessed a clustered population structure compared to the tide pool subpopulation with a mild environment. This may be due to the extreme environment of the rock microhabitat selecting for non-neutral loci, and the death of heat-intolerant rock individuals after being exposed to high summer temperatures, allowing non-adapted alleles to be removed from the survivors on the rocks, which may increase the frequency of heat-tolerant genes in the entire population. This suggests that natural selection plays a major role in the construction of neutral genetic diversity patterns.^71^^,^^72^ It can be hypothesized that the persistence of rapid adaptive selection is crucial for the survival of organisms in sun-exposed microhabitats.

### Benign microhabitats as refugia against thermal stress

Tidal pools can provide relatively unstressed environmental conditions and shelters for organisms in the intertidal zone.^73^ Importantly, benign habitats such as tidal pools have been shown to provide refuge for organisms against thermal stress.^30^^,^^43^^,^^74^^,^^75^ In the present study, tidal pools were found to be less thermally stressful than emergent rocky microhabitats, thereby offering refugia to intertidal organisms. Benign microhabitats have also been found to preserve physiological and genetic polymorphisms in times of extreme environmental stress, avoiding the extinction of organisms.^34^^,^^76^ Furthermore, such benign environments are more predictable than harsh ones.^30^ The presence of small-scale microhabitats with large temperature differences allows organisms to maintain a wide range of distribution at latitudinal scales.^77^ Therefore, the heterogeneity of intertidal microhabitats should be taken into account when studying the physiological and evolutionary adaptations of intertidal organisms and assessing and predicting their thermal sensitivity to climate change.

Another advantage of tide pool microhabitats in bolstering organismal resilience to environmental stresses lies in the uninterrupted availability of oxygen. In rocks microhabitats, mussels adopt a strategy of shell closure during air exposure to mitigate water loss. However, the seawater retained within the mussel’s shell contains only limited dissolved oxygen, depleting rapidly and necessitating a transition to anaerobic metabolism.^65^^,^^78^ This shift from aerobic to anaerobic metabolism is frequently accompanied by a precipitous decline in cardiac activity, accounting for the lower heart rate observed in aerial temperature ramps.^79^ In contrast, mussels inhabiting tide pool microhabitats benefit from a consistent supply of oxygen within the water column, affording robust protection against environmental warming.

### Conclusion

Environmental complexity across microhabitats must be taken into consideration when predictions are made of how biota will respond to future climate change. Relatively benign microhabitats can provide a buffer against extinction or range shifts due to climate change. Rapidly generated differences at the genetic level in conspecifics occurring across small-scale microhabitats with different physical conditions can lead to different molecular responses, e.g., gene expression and RNA editing, and physiological phenotypes, e.g., cardiac thermal sensitivity. These genetic and physiological adaptations across microhabitats are apt to be highly important for preventing catastrophic biological events in the face of climate change. Therefore, when using temperature sensitivity to predict the effects of climate warming, it is essential to take into account all aspects of adaptive variation across microhabitats.

### Limitations of the study

While our results showed that the subpopulations in the tidal pool and on emergent rocks had different genetic structures and exhibited different physiological and molecular responses to high-temperature stress, several limitations should be acknowledged. Firstly, our study employed RNA-seq to assess genetic differentiation in two microhabitats, which can only capture genetic disparities at coding genes. Secondly, it is important to note that genetic structure, molecular responses, and physiological traits within microhabitats may exhibit seasonal variations. Finally, the differences among microhabitats could also be influenced by a multitude of environmental factors (e.g., dissolved oxygen and pH) and biotic factors (e.g., predation and competition).

## STAR★Methods

### Key resources table

**Table:** 

REAGENT or RESOURCE	SOURCE	IDENTIFIER
**Deposited data**		

RNA-seq data	This study	SRA (BioProject ID: PRJNA938804; BioSample accessions: SRR23682408 - SRR23682361)
Original code	This study	https://figshare.com/s/2b45e174022b4be95d40

**Software and algorithms**		

R softwares	R project	https://www.r-project.org/
FastQC	Andrews et al.^80^	https://github.com/s-andrews/FastQC
fastp	Chen et al.^81^	https://github.com/OpenGene/fastp
HISAT2	Kim et al.^82^	https://github.com/DaehwanKimLab/hisat2
Samtools	Li et al.^83^	https://github.com/samtools/samtools
StringTie	Pertea et al.^84^	https://github.com/gpertea/stringtie
DESeq2	Love et al.^85^	https://github.com/thelovelab/DESeq2
bwa mem	Li et al.^86^	https://github.com/lh3/bwa
REDItools	Picardi et al.^87^	https://github.com/BioinfoUNIBA/REDItools
GATK	McKenna et al.^88^	https://github.com/broadinstitute/gatk
HaplotypeCaller	Poplin et al.^89^	https://github.com/BGI-biotools/HaplotypeCaller-Acc
VCFTools	Danecek et al.^90^	https://github.com/vcftools/vcftools
PLINK	Purcell et al.^91^	https://github.com/insilico/plink
ADMIXTURE	Alexander et al.^92^	https://github.com/azzaea/admixture
FastTree	Price et al.^93^	https://github.com/PavelTorgashov/FastTree
poppr package	Kamvar et al.^94^	https://github.com/grunwaldlab/poppr

**Other**		

Fluke Infrared camera	Fluke Corporation	TiX660
Robomussel	data logger iButton	DS1922L; Maxim Integrated

### Resource availability

#### Lead contact

Further information and requests for resources and reagents should be directed to and will be fulfilled by the lead contact, Yun-Wei Dong (dongyw@ouc.edu.cn).

#### Materials availability

This study did not generate new unique reagents.

#### Data and code availability

• The RNA-seq raw data have been deposited at the Sequence Read Archive (SRA) and are publicly available as of the date of publication. The accession number is listed in the key resources table.
• All original code has been deposited at FigShare online and is publicly available as of the date of publication. The url link is listed in the key resources table.
• Any additional information required to reanalyze the data reported in this work paper is available from the lead contact upon request.

### Experimental model and subject details

This study was based on wild mussel *Mytilisepta virgata*, and no experimental models were used.

### Method details

#### *In situ* temperature measurement

The operative temperatures of *Mytilisepta virgata* exposed to different environmental conditions were continuously recorded using biomimetic thermal loggers, Robomussels, under both immersed and emersed conditions.^34^ For preparing a Robomussel, a data logger iButton (DS1922L; Maxim Integrated) was inserted into an empty shell of a mussel (shell length, 4–5 cm), which then was filled with 3M Scotchcast 2130 Flame Retardant Compound.^95^ The Robomussel simulates the thermal buffering of an actual *M. virgata* mussel to more accurately estimate the temperature changes observed by individuals observed in this study. These Robomussels were deployed in tidal pools (30–50 cm in depth) and the emergent south-facing rocks in the intertidal zone of Dongshan (23.65°N, 117.49°E), Fujian Province, China from July to September 2020. The measurement accuracy of the iButton was set to 0.5°C with a sampling interval of 20 min. As described in a previous study,^96^ the ninety-ninth percentile temperature of each month (T_99_) was considered as the acute thermal stress temperature of *M. virgata* in that month; T_99_s were calculated for tidal pool and rock microhabitats separately. To characterize further the different thermal properties of tidal pool and emergent rocks microhabitats, an infrared thermal image analysis was conducted by using a Fluke TiX660 Infrared camera (Fluke Corporation, USA).

#### Animal collection and acclimation

The mussels with a similar size (shell length, 3-4cm) were randomly collected in tidal pool and rock microhabitats in Dongshan, Fujian, China, and were transported to the laboratory at Dongshan Swire Marine Station (D-SMART) within 1 h. The mussels were collected from both tidal pools (31 rock individuals) and emergent rocks within 20 cm (32 tidal pool individuals). All specimens were acclimated under immersion for 2 days at a water temperature of 25°C and salinity of 28 psμ prior to use in the experiments described below. The temperature of 25°C was chosen as it represents the average annual temperature in Dongshan where the mussels were collected. The salinity level of 28 psμ was selected to align with the typical seawater salinity range of the mussel’s habitat. Additionally, a two-day acclimation period was established, to remove the effects of sampling and transportation.

(1) Aerial temperature ramps: Mussels were heated from 25 °C at a rate of 0.1°C min-1 in the air until the designated temperature (30, 35 or 43°C) was reached. To remove the effect of the length of heating time on the results, for the 25°C groups, mussels were held at 25°C for a whole exposure period (∼3.5 h). For the 30, 35°C and 43°C groups, mussels were maintained at the designated maximal temperatures for different durations to maintain the same exposure periods (∼3.5 h) that simulated thermal stress in the natural environment.
(2) Immersion temperature ramps: Animals were placed into beakers with seawater (25°C, 28 psμ). The water temperature was increased to the designated temperatures at a rate of 0.1°C min-1 and then maintained for different durations to maintain the same exposure periods of 3.5 h.

#### Heart rate measurement and analysis

A non-invasive method was used to measure individual heart rates.^97^ An infrared sensor (IR-EX; Newshift, Portugal) for heart rate measurement was directly attached next to the mid-dorsal posterior hinge area of the valves using Blu-Tack (Bostik).^98^ None of the black mussels exhibited mortality during the experiment.

An infrared sensor (CNY70) was fixed directly above the heart using Blue-Tack (Bostik, United Kingdom). Variations in the light-dependent current produced by the heartbeat were amplified, filtered and recorded using an infrared signal amplifier (AMP03, Newshift, Portugal) and PowerLab AD converter (8/30, ADInstruments, Australia). Data were viewed and analyzed using LabChart v7.2 (ADInstruments, Australia). Real-time heart rates were recorded every minute.

#### Transcriptomic responses to heat stress

After the heating treatment, the gill tissue of each sample was dissected and placed into a 2 mL tube with 1 mL of RNAlater solution (Thermo Fisher Scientific). Tubes were stored overnight at 4°C to allow the RNAlater solution to inhibit RNase. Samples were then transferred to a −80°C freezer for storage for transcriptome sequencing experiments.

Total RNA was extracted with an EZNA Mollusc RNA kit (Omega Bio-Tek, USA) following the manufacturer’s instructions. The concentration and integrity of column-purified RNAs were determined using NanoDrop spectrophotometry (ND-1000) and 1% agarose gel electrophoresis, respectively. The RNA samples used in the present study showed high purity (i.e., A260/230 and A260/280 ratios > 1.8) and integrity (i.e., tight 18S and 28S ribosomal RNA bands). A total of 63 RNA samples (two microhabitats × seven treatments × three-five replicates) were subjected to standard library construction procedures using the NR604-VAHTS Universal V6 RNA-seq Library Prep Kit from Illumina (Vazyme, Nanjing, China) according to the manufacturer’s protocol. Library quality was assessed using a 2100 Bioanalyzer (Agilent Technologies). The library preparations were sequenced on an Illumina HiSeq 6000 platform (BerryGenomics Co., Ltd., Beijing, China) and 150-bp paired-end reads were generated.

The RNA-seq data are available in the NCBI short-read archive database (BioProject ID: PRJNA938804; BioSample accessions: SRR23682408 - SRR23682361). The quality control assessment was performed using FastQC^80^ version 0.11.9; adapters, low-quality bases (Q < 20), and shorter reads (<25 bp) were removed using fastp^81^ version 0.23.2 for cleaning data. The filtered reads were mapped to the *M. virgata* genome (BioProject: PRJNA910323) by HISAT2^82^ version 2.2.1. The alignment files were then compressed, sorted and indexed by Samtools version 1.15.1.^83^ The gene expression levels were quantified by using StringTie version 2.0 with default options.^84^ Read data for differential expression analysis were obtained from the python script “prepDE.py” provided by the StringTie^84^ authors (http://ccb.jhu.edu/software/stringtie/dl/prepDE.py3). Differential expression analysis was performed using DESeq2^85^ version 1.28.1 in the Bioconductor package, in both immersion and aerial temperature ramps, temperature was used as an additional variable in a multi-factor design using DESeq2 to obtain differentially expressed genes between the microhabitats across the temperature. Genes were considered differentially expressed when log2|fold change| > 1 and adjusted p value <0.05. The volcano plot of differentially expressed genes (DEGs) was drawn using the ggplot2 R package. DEGs were considered to be significantly enriched in GO terms when the p value was <0.05. The Kyoto Encyclopedia of Genes and Genomes (KEGG) pathway enrichment analysis was used to gain insights into the functional significance of the changes in gene expression.^99^ ClusterProfiler v4.0.2 R package was used to determine the KEGG pathway for the enrichment of DEGs. A heatmap of KEGG-enriched pathways in the associated DEGs was drawn using pheatmap version 1.0.12 R package (https://cran.r-project.org/web/packages/pheatmap/).

#### Neutral genome-wide variation analysis

Raw reads were trimmed and assessed for quality control using fastp,^81^ before alignment with bwa mem.^86^ Aligned SAM files were processed with Picard SortSam and MarkDuplicates to remove PCR duplicates and allow conversion to BAM files. Sorted BAM files were used for identifying edited sites using the REDItools^87^ version 2.0. BAM files were used for SNP calling with GATK^88^ and HaplotypeCaller^89^ against the reference genome (https://github.com/broadinstitute/gatk). The GATK VariantFiltration standard hard filtering option (‘QUAL <30.0 || QD < 2.0 || FS > 60.0 || SOR >4.0’) was used to filter SNPs. The VCF file containing high-quality SNPs was filtered based on the following criteria using VCFTools version 0.1.16^90^: all SNPs with >50% missing data, SNPs with quality score <30, and SNPs with a minor allele count of 3 or less were removed (--max-missing 0.5 --minQ 30 --mac 3). Quality filtration was performed on these markers using PLINK version 1.90.^91^ Minor allele frequency (MAF) less than 5% (--maf 0.05), markers with more than 10% absence (--geno 0.1), and individuals with more than 10% missing SNP calls (--mind 0.1) were considered for filtration, and within Hardy-Weinberg equilibrium (HWE) at p < 0.005 (--hwe 0.005). Then, the --indep-pairwise 50 10 0.5 commands in PLINK were used to calculate pairwise LD within a 50 SNP window and remove one SNP from a pair where the LD exceeds 0.5, before moving on 10 SNPs and repeating the procedure. A total of 220,762 SNPs filtered for HWE and LD were determined as “neutral SNPs”.

#### Non-neutral genome-wide variation analysis

To assess whether non-neutral genomic variation occurs within the microhabitat, the loci with Fst >0.15 (indicating significant population difference^100^) were identified as outliers. Initially, an “overall SNPs” dataset of 7,990,053 was created using GATK. With site missing data >50%, quality score <30, minor allele frequencies of 0.05, minor allele count of 3 or less, MAF <5%, absence less than 10%. Fst to calculate the genetic distance between populations was determined using vcftools -weir-fst-pop option, then outliers were identified from the overall SNPs with Fst >0.15.

#### Genetic structure and heterozygosity

Population structure analyses were performed using ADMIXTURE^92^ version 1.3.0, then run with default values for multiple values of K, and the resultant admixture profiles were plotted to determine the optimal K value. Principal component analysis (PCA) was performed using PLINK. The approximate maximum likelihood tree was constructed with FastTree using the generalized time-reversible model,^93^ visualized the evolutionary tree with itool (https://itol.embl.de/). Evidence for population genetic structure was assessed using Analysis of Molecular Variance (AMOVA) that was conducted with the poppr.amova function as implemented in the poppr package.^94^ And heterozygosity was calculated using the PLINK version 1.90 (--het).^91^

#### RNA editing analysis

The data employed for the SNP analysis was also used for the RNA editing analysis. We quantified RNA editing levels of a total of 63 individuals across the two microhabitats. Editing levels of a site were calculated by determining the fraction of reads with a ‘G’ nucleotide at that site. Clean data were obtained by quality control of the raw data using FastQC^80^ version 0.11.9 and fastp^55^ version 0.23.2. Based on the aligned BAM files, we used REDItools2^87^^,^^101^ with the parameters “reditools.py -f $i.sort.bam -o -S -s STRAND” to identify RNA editing sites (RESs) in the mRNA sequencing (RNA-Seq). REDItools2 is a software package for genome-wide RNA editing site detection with prealigned reads in Binary Alignment/Map (BAM) format as input. REDItools2 can be used alone with RNA-Seq data for editing site detection. In the case of RNA-Seq data, only those editing sites that are statistically significant with the empirically observed distribution of base substitutions are identified as RNA editing events. In addition, REDItools2 implements various filters and quality checks to eliminate potential false positives. For each mussel line of microhabitats in treatments with different temperatures, the editing levels of each site were averaged between at least three biological replicates. We only compared sites that had at least 50 reads in both biological replicates and had less than 20% difference in editing between replicates in at least three samples in each group being compared, i.e., the RNA editing sites with greater than 20% difference between biological replicates in the same group was filtered out, and the RNA editing sites which account for only 2% or less of all 63 individuals were also eliminated.^102^

#### Statistical analyses

The differences in heart rate of mussels from different microhabitats and the difference in RNA editing level of mussels in different microhabitats were analyzed using generalized linear model (GLM) with a Gaussian model in SPSS 22 (SPSS, Chicago, IL, USA). In light of the nonnormal distribution inherent in our factor data and the imperative to account for potential interactions among factors, the adoption of the GLM emerges as a judicious choice.^103^

### Quantification and statistical analysis

All quantification and statistical analyses were performed as described in the method details section of the STAR methods.
