# Supplementary material for: The impacts of thermal heterogeneity across microhabitats on post-settlement selection of intertidal mussels
Source: iScience. 2023 Oct 31;26(12):108376. doi: 10.1016/j.isci.2023.108376 (PMC10682278; doi:10.1016/j.isci.2023.108376)
Supplement: Document S1. Figures S1, S2, and Table S1 [file mmc1.pdf]

iScience, Volume *26*

Supplemental information

The impacts of thermal heterogeneity across microhabitats on post-settlement selection of intertidal mussels

Yue Tan, Yong-Xu Sun, Ya-Jie Zhu, Ming-Ling Liao, and Yun-Wei Dong

Table S1. Analysis of molecular variance (AMOVA) showing the genetic variance among and within the rock and tidal pool microhabitat of non-neutral loci, Related to STAR Methods.

	**Source**	**df**	**SS**	**MS**	***P***	**Variance (%)**
non-neutral genetic variation	Between microhabitat	1	23943.29	23943.29	0.001	13.74
	Within microhabitat	61	242695.20	3978.61	0.001	86.26
	Total	62	266638.49	4300.62		100

Df = degrees of freedom; SS = sum of squares; MS = mean squares

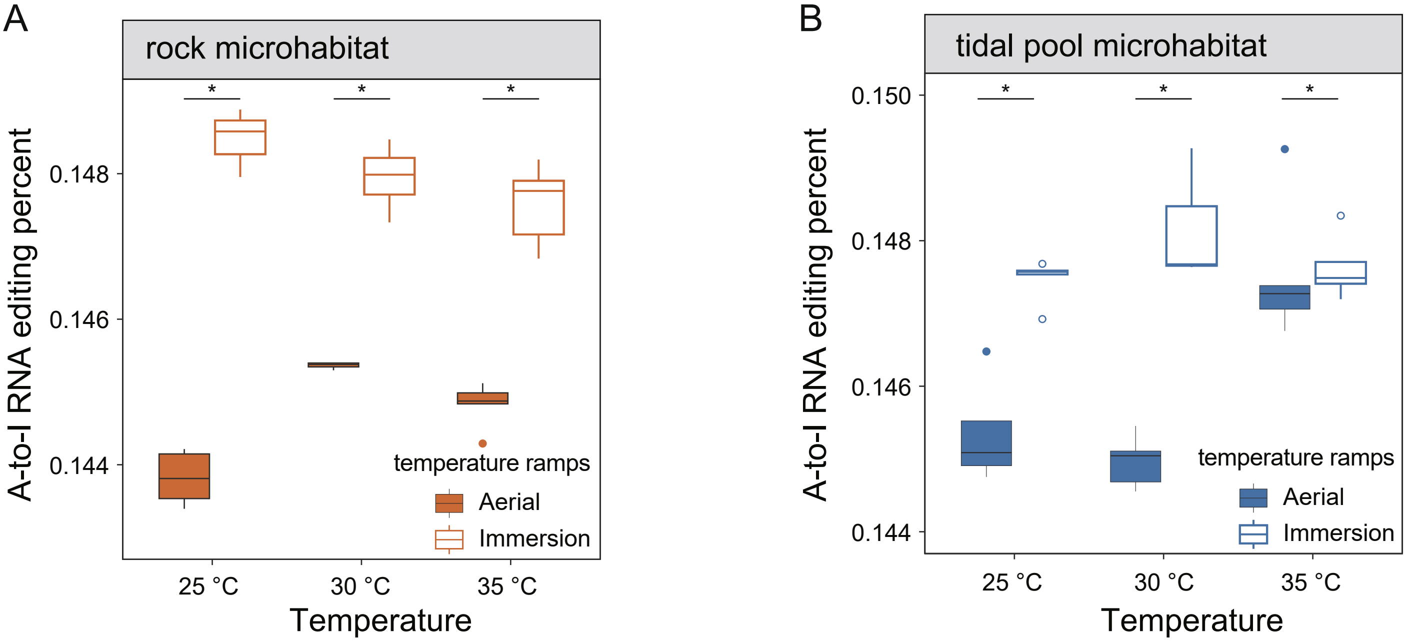

Figure S1 RNA editing analysis of mussels in different treatment groups (A) The proportion of A-to-I RNA editing of mussels under aerial and immersion treatment in rock. (B) The proportion of A-to-I RNA editing of mussels under aerial and immersion treatment in tidal pool. Asterisk denotes statistically significant differences * $p<0.05$. (GLM).

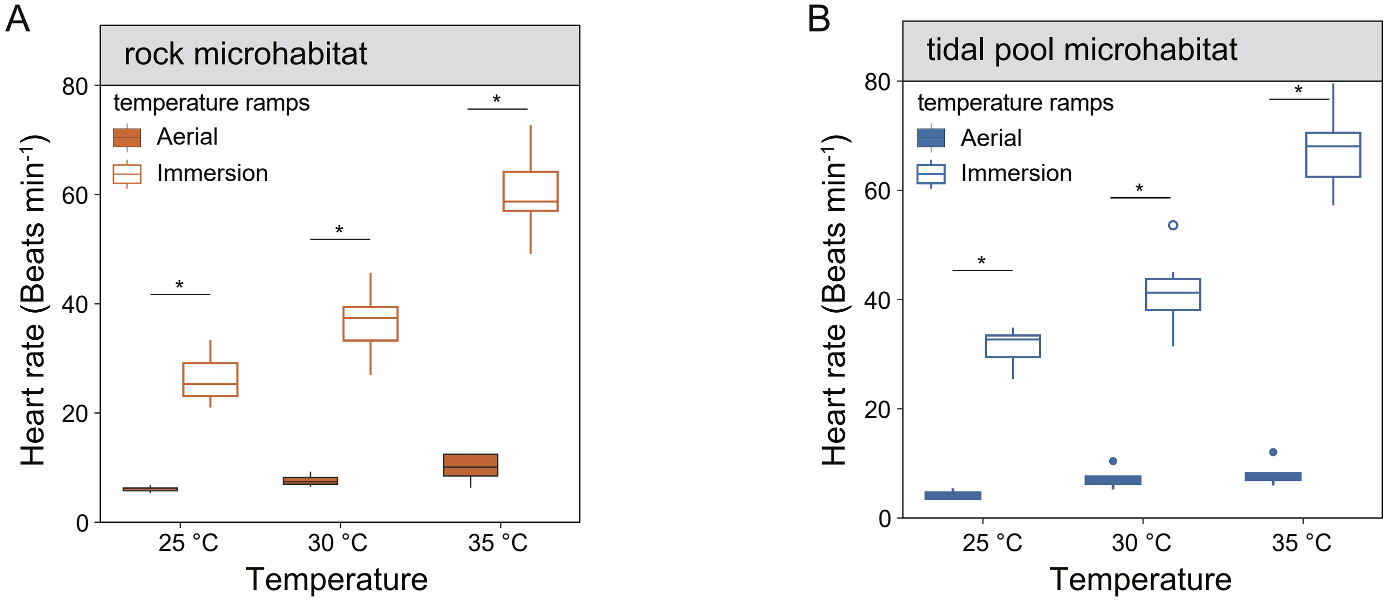

Figure S2 Cardiac performance of mussels in different treatment groups (A) The cardiac performance of mussels under aerial and immersion treatment in rock. (B) The cardiac performance of mussels under aerial and immersion treatment in tidal pool. Asterisk denotes statistically significant differences * $p<0.05$. (GLM).
